# Effects of Acute Exercise on Drug Craving, Self-Esteem, Mood, and Affect in Adults with Polysubstance Use Disorder: Protocol for a Multicenter Randomized Controlled Trial

**DOI:** 10.2196/18553

**Published:** 2020-10-13

**Authors:** Maren Mikkelsen Ellingsen, Sunniva Launes Johannesen, Egil Wilhelm Martinsen, Sandra Rinne Dahl, Mats Hallgren

**Affiliations:** 1 Department for Inpatient Treatment of Substance Misuse Division of Mental Health and Addiction Oslo University Hospital Oslo Norway; 2 Institute of Clinical Medicine University of Oslo Oslo Norway; 3 Hormone Laboratory Department of Medical Biochemistry Oslo University Hospital Oslo Norway; 4 Department of Public Health Sciences Karolinska Institutet Stockholm Sweden

**Keywords:** exercise, acute, substance abuse, physical activity, drug addiction

## Abstract

**Background:**

Novel treatments for substance use disorders are needed. Acute bouts of exercise can improve mood states and craving in nonclinical populations. Exercise effects in those with polysubstance dependence are understudied; controlled trials are needed.

**Objective:**

This protocol describes a clinical study examining the short-term psychological effects of 2 types of physical activity, soccer and circuit training, in patients with substance use disorders. Effects will be compared with a nonexercise control group. Specific aims are to investigate whether there are differences between the activities and the duration of changes.

**Methods:**

This study is a short-term multicenter randomized control trial with a crossover design. Patients consecutively admitted to 4 inpatient treatment centers were invited to participate in 3 conditions, each lasting 45 minutes, within one week. The order of the conditions was randomized. There were a total of 5 assessments, taken at baseline, immediately before each condition, immediately after each condition, and 1, 2, and 4 hours postintervention, enabling patterns of change over time to be observed. Psychological effects were assessed with self-report questionnaires, which included scales for craving, state anxiety, positive and negative affect, self-esteem, and mood. Exercise intensity was assessed with the Borg Rating of Perceived Exertion scale and a heart rate monitor (Polar M200; Polar Electro Ltd). Cortisol was assessed in saliva before and 4 hours after the intervention.

**Results:**

A total of 39 patients were included in the study. Data collection was completed in 2019.

**Conclusions:**

We anticipate larger improvements in the intervention groups than among controls, indicating positive psychological effects during and after exercise. The study will add clinically relevant information about the short-term psychological effects of exercise in the treatment of substance use disorders, using activities that are easily accessible in different clinical settings.

**Trial Registration:**

German Clinical Trials Register DRKS00018869; https://www.drks.de/drks_web/navigate.do?navigationId=trial.HTML&TRIAL_ID=DRKS00018869

**International Registered Report Identifier (IRRID):**

DERR1-10.2196/18553

## Introduction

Substance use disorders (SUD) have wide-reaching impacts on health and well-being and contribute substantially to the global disease burden [1]. They are associated with shorter life expectancy, and comorbid medical conditions are common [2]. The prevalence of co-occurring personality and other psychiatric disorders is high, with estimates ranging from 50% to 90% [3]. Mood and anxiety disorders are prevalent and affect both the severity and outcome of treatment [4]. Current treatment options for SUDs include cognitive behavioral therapy, motivational interviewing, and medication. Although documented as effective, these methods do not help all patients. Relapse rates range between 40% and 60% [5], and dropout rates from in-patient treatment is around 30% [6].

Despite their high prevalence, few people with SUDs ever receive treatment, and there is a need for more treatment options [7,8]. One potential explanation for poor help seeking is the perceived stigma associated with traditional treatments [9,10]. Lifestyle-focused interventions have potential to increase help seeking by reducing this stigma.

Physical activity has been suggested as an alternative or complementary treatment option for SUDs [11-13]. The rationale is that physical exercise might be effective due to its beneficial effects on factors related to relapse and the maintenance of the disorder, such as comorbid mental disorders, craving, and emotional dysregulation [14-17].

Regular physical activity increases fitness and lowers the probability of chronic diseases [18,19]. It has been proposed as a strategy for alleviating symptoms of psychiatric disorders in general [20,21], and its application in the mental health care system is increasing. Regular physical activity has been shown to be effective in the prevention and treatment of common mental health conditions [22]. Studies also indicate that physical activity alleviates symptoms in people suffering from severe mental disorders [23]. Acute bouts of exercise have cognitive and mood-enhancing benefits, including improved executive functioning and lower state anxiety and stress reactivity [24,25]. The mechanisms behind the psychological benefits of exercise in SUDs are not well established, but one tenable hypothesis is that they are partly mediated by changes in the stress hormone cortisol [24].

While the effects of exercise are well documented for common mental disorders, there has been less research focus on SUDs. Most studies have addressed the effect of chronic exercise interventions on smoking cessation or alcohol misuse. Long-term exercise programs might affect smoking behavior [26] and lower tobacco cravings [27]. A meta-analysis of 21 exercise studies for alcohol use disorder concluded that, while regular exercise did not seem to affect consumption, it had beneficial effects on depression and physical fitness [28]. In another meta-analysis, Wang et al [29] found that exercise was associated with greater abstinence rates among illicit drug users compared with those using alcohol and nicotine. The effect of exercise on withdrawal symptoms, anxiety, and depression was not moderated by the type of substance used. Colledge et al [30] reviewed the effects of anaerobic exercise on SUDs. The results were mixed, with some evidence of a positive effect on abstinence from nicotine. No conclusions could be drawn regarding the effects of exercise among illicit drug users.

One plausible explanation for the beneficial long-term effects of an exercise program is that many single sessions have a cumulative long-term benefit. Few studies have addressed the short-term effects of physical activity in SUDs. A study of 45 regular smokers found that bouts of moderate and vigorous exercise provided relief from withdrawal symptoms, while moderate exercise relieved distress and improved mood [31]. Studies addressing alcohol urges or cravings show promising results, with lower craving after low-to-moderate intensity exercise [13,32]. Wang et al [33] studied the development of craving for amphetamines and found a reduction in cravings during and immediately after stationary cycle exercise. Results suggest that the intensity of the exercise may affect craving, with the lowest craving following moderate-to-vigorous exercise [34].

The misuse of a single substance is exceptional in SUDs [35]. Whether acute exercise can benefit people with polysubstance dependencies is unclear. Most studies have assessed aerobic forms of exercise, often in laboratory conditions using stationary bicycles [12,28]; little is known about other forms of exercise in clinical settings. With some exceptions [25], psychological benefits across different forms of exercise have not been compared, and it is unclear how long the changes last following a single exercise session. Studies have mostly used pre- and postassessments only, which is a limitation.

To address these questions, we conducted a feasibility study to compare the short-term effects of 3 different exercises (soccer, circuit training, and walking) conducted in nonlaboratory settings [36]. Findings suggested that moderately intense exercise activities performed in natural settings may increase mood, help attenuate drug cravings, increase positive affect, decrease negative affect, and improve self-esteem. The results were promising but need to be replicated with a larger sample and a randomized design.

In this study, we are conducting a multicenter randomized controlled trial to compare the short-term effects of 2 forms of exercise (soccer and circuit training) with a control condition (a lecture on the health benefits of physical activity) in polydrug-dependent inpatients. We chose to compare the effects of exercise to a nonexercising control group (lecture) instead of a walking-based intervention, as previous studies have shown that even light physical activity can have mood-enhancing effects [37]. We are also performing biomarker analyses of cortisol to better understand the underlying mechanisms.

Key research questions that will be addressed include (1) What are the short-term effects of soccer and circuit training on mood (primary outcome), drug craving, positive and negative affect, state anxiety, and self-esteem in adults with SUD? (2) How long do the effects last after the exercise sessions end? (3) Are there differences between the two forms of exercise? (4) Do the effects differ with the intensity of the exercise? and (5) Are the study outcomes associated with changes in the stress hormone cortisol?

Based on previous studies [33,38], we hypothesize that there will be positive psychological effects on the study outcomes after exercise, with a reduction in craving and an improvement in mood and affects. We expect to find larger improvements in the intervention groups than in the control group.

## Methods

We adhered to the SPIRIT (Standard Protocol Items: Recommendations for Interventional Trials) guidelines in the preparation of this protocol. The study will be reported according to CONSORT (Consolidated Standards of Reporting Trials) ethical guideline recommendations.

### Ethics Approval and Consent to Participate

The trial is approved by the Regional Committee for Medical and Health Research Ethics in South East Norway (2018/1275) and was retrospectively registered with the German Clinical Trials Register on November 11, 2019 (DRKS00018869). Before inclusion in the study, participants were asked to sign an informed consent form. All patients agreed to participate voluntarily and were told they were free to withdraw from the study at any time.

### Setting and Participants

Inpatients from 4 treatment facilities were invited to participate. The main site for the study was the Department of Addiction Treatment - Adult, Division of Mental Health and Addiction, Oslo University Hospital. In addition, 3 other treatment centers were asked to participate (2 accepted): the Department of Addiction Treatment - Youth, Oslo University Hospital; an inpatient treatment center at the Division of Mental Health and Addiction, Vestfold Hospital Trust; and the Blue Cross Treatment Center, Slemdal, Oslo. The study was conducted in a clinical setting, where the participants received treatment.

The treatment centers are staffed by medical doctors, nurses, psychologists, and social workers. Treatment includes individual and group counseling and pharmacotherapy. Patients participate in structured group sessions and practice activities of daily living, including routines for house cleaning, eating, and sleeping. They receive individual sessions with psychologists, doctors, and social workers and participate in team meetings and social training. Physical exercise, including strength training, ball games, walks, and running, are part of the usual treatment program. Before admission to treatment, all patients complete detoxification.

Recruitment took place at the treatment centers, where patients were given information and invited to participate in the study by members of the research group and a local project coordinator. All participants were diagnosed with a SUD based on the International Classification of Diseases, Tenth Revision (ICD-10), were inpatients at one of the treatment centers, and were 18 years or older.

### Study Design and Randomization

This study is a short-term, multicenter randomized controlled trial with a single pretest and multiple posttests. Using a crossover design, changes in mood, craving, state anxiety, affect, and self-esteem were assessed by comparing the initial measurement (taken immediately before exercise) with 4 follow-up assessments, first immediately after exercise, then at 1, 2, and 4 hours postintervention. The design allows the pattern of change over time to be seen.

Participants completed 3 sessions—2 supervised group exercise sessions (soccer and circuit training) and 1 control condition (attending a lecture about the health benefits of physical activity)—within the same week. All the sessions were performed in a group setting.

The order of the study conditions was determined by a random number generator and placed in sealed envelopes at a site separate from the study location. When a group of suitable patients (5-10 patients) was recruited at a site, a sealed envelope with the order of exercise and control conditions was drawn by a person outside of the research group.

### Procedure

Patients were invited to attend an information meeting, where members of the research group explained the purpose of the study in detail and handed out written information. To minimize dropout, the recruitment took place a maximum of two weeks ahead of the trial. A member of the research group visited the treatment center 1 week before the study to answer questions.

After giving informed consent, eligible participants were gathered to perform the interventions and assessments. These sessions were undertaken on-site at the clinics starting at 9:30 AM on Monday, Wednesday, and Friday within one week. The participants were asked to refrain from doing exercise on the days of the trial and on the days in between the interventions to give a 1-day washout period between the sessions. All sessions lasted 45 minutes. During the week of the trial, all participants received treatment as usual between the sessions.

The following exercise activities and control condition were tested in random order: (1) indoor soccer, (2) circuit training in a gymnasium using body weight, and (3) the control condition, attending a lecture on exercise and mental health.

**Figure 1:**
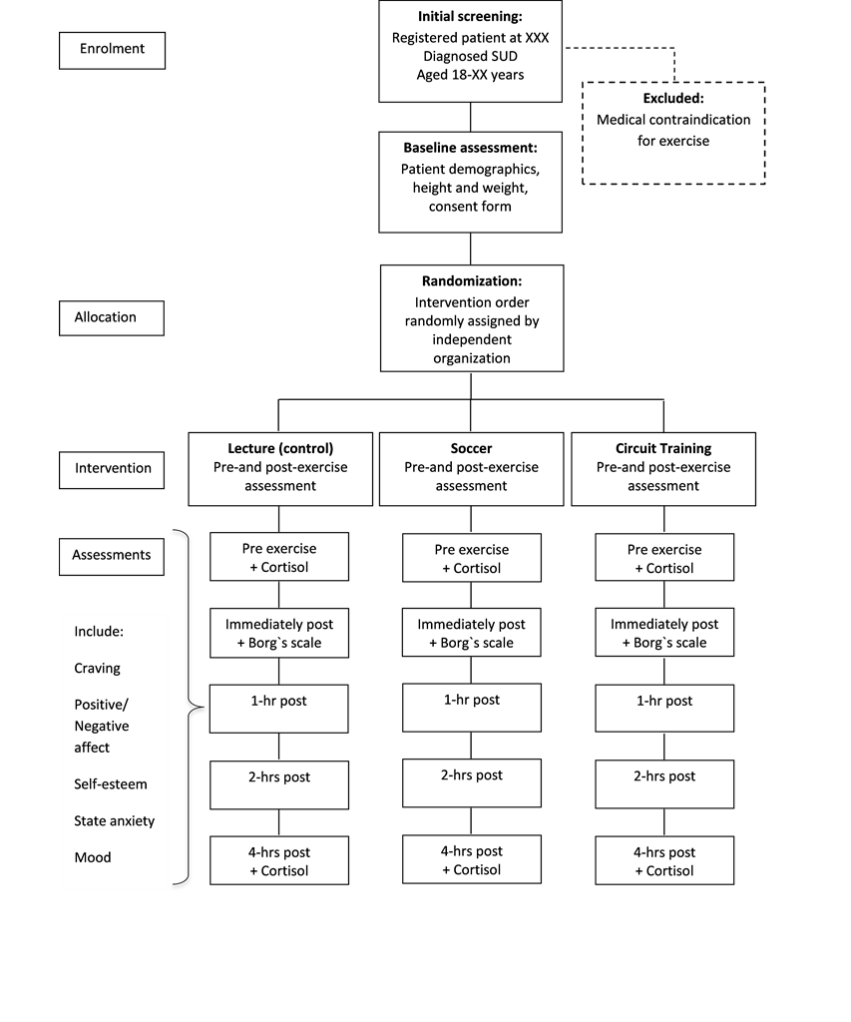
Flow of participants through the study. SUD: substance use disorder.

Across all trial days, the participants were advised to eat breakfast about an hour before starting the trial. They were advised to have a light meal or snack after training, have lunch on all trial days, and keep hydrated throughout the day. Participants were also asked to limit the use of caffeine and nicotine during the test period. The flow of participants through the trial is shown in Figure 1.

### Exclusion Criteria

Patients admitted to the treatment centers typically have comorbid ICD-10 mental disorders. None of these served as exclusion criteria as long as the participant was able to give informed consent. All patients at the clinics were invited to join; the only exclusion criterion was having a physical condition or injury that would impede physical activity participation. Some patients relapsed to substances during treatment. Patients who were suspected to be under the influence of drugs were asked to discontinue.

### Measures

#### Background Information

Therapists collected background information about the participants during an interview. The therapists filled in a questionnaire covering sociodemographic and treatment-related variables, ICD-10 substance use and mental disorders diagnosed during treatment, duration of problematic drug use, treatment history, level of activity before admission, and attitude toward physical activity. Height and weight were recorded. For most participants, this was based on measures taken at the treatment centers. A minority were self-reported.

#### Baseline and Follow-up Assessments

Mood (primary outcome), craving, affect, self-esteem, and state anxiety were measured with self-report questionnaires. The same questionnaires were repeated for baseline and follow-up assessments. The questionnaires chosen could be completed in 5 minutes, were amenable to repeated administration, and had sound psychometric properties.

**Figure 2:**
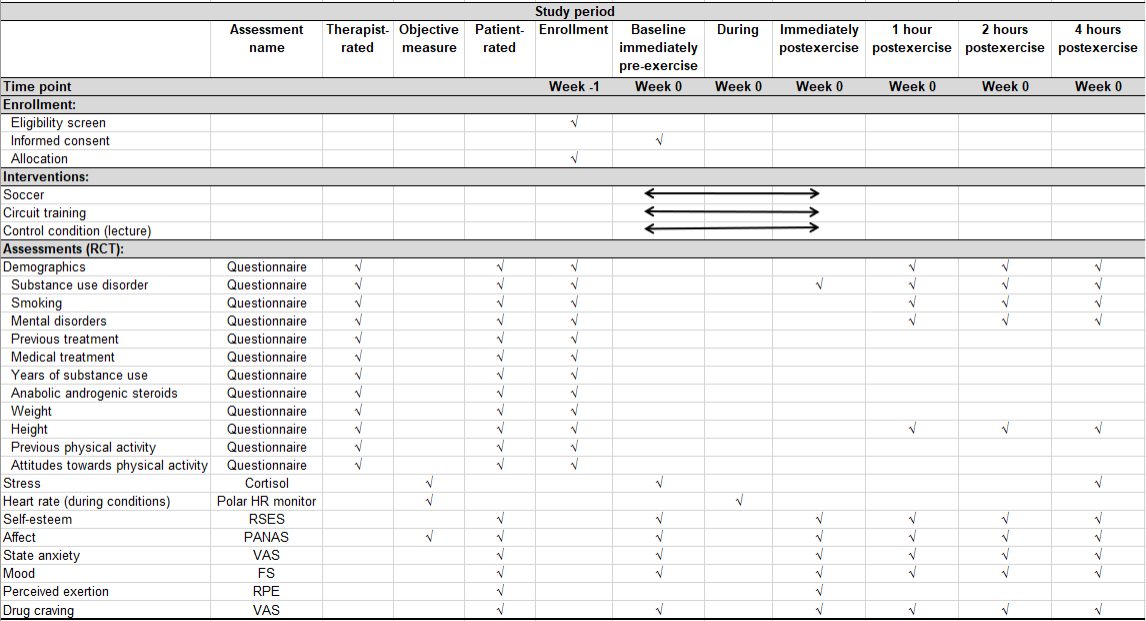
SPIRIT figure, study measures, and assessment time points. FS: Feeling Scale; HR: heart rate; PANAS: Positive and Negative Affect Schedule; RCT: randomized controlled trial; RPE: Rating of Perceived Exertion; RSES: Rosenberg Self-Esteem Scale; VAS: visual analog scale.

The self-report questionnaires and saliva samples collected immediately before the sessions were the baseline assessment. Following the sessions, participants filled in self-report questionnaires on 4 occasions: immediately after the session and 1, 2, and 4 hours after the session. Immediately after the exercise sessions, participants were also asked to rate the perceived intensity. A saliva sample was gathered 4 hours after all sessions. Figure 2 illustrates the SPIRIT figure, study measures, and assessment time points.

Self-report measures were (1) the Feeling Scale, (2) drug craving, (3) the Positive and Negative Affect Schedule (PANAS), (4) the Rosenberg Self-Esteem Scale, (5) state anxiety, and (6) the Borg Rating of Perceived Exertion (RPE) scale.

First, changes in mood related to exercise were assessed with the Feeling Scale [39] (primary outcome), a single-item Likert scale ranging from –5 (very bad) to 5 (very good). Participants were instructed to indicate how they felt “at this moment.” The scale has been used extensively in acute exercise studies [40].

Second, drug craving was assessed using a single-item visual analog scale (VAS) ranging from 0 (no craving for drugs) to 10 (strong craving for drugs). Participants were instructed to indicate how strong their craving for drugs felt “at this moment.” The instrument has been used in previous studies of acute exercise [41] and it was tested in our recent pilot study [36], where it was shown to be sensitive to change.

Third, the PANAS assesses 2 dimensions of affect [42]. Participants self-rate 20 items on a 5-point Likert scale ranging from 1 (very slightly/not at all) to 5 (extremely). Ten of the items represent positive affect and the others represent negative affect.

Fourth, self-esteem was measured using the 10-item Rosenberg Self-Esteem Scale [43], which measures global feelings about oneself (that is, both positive and negative feelings). All items are answered using a 4-point Likert scale ranging from strongly agree to strongly disagree.

Fifth, state anxiety was assessed by a single-item VAS-scale ranging from 0 (no anxiety) to 10 (intensive anxiety/full panic).

Sixth, the Borg RPE scale [44] was administered 5 minutes after each exercise session to assess how strenuous the exercises were perceived by participants. The single-item scale ranges from 6 (no exertion at all) to 20 (maximal exertion).

#### Device-Based Assessment of Physical Activity

In addition to collecting self-reports on how strenuous the exercises were perceived to be, data on the average and maximum heart rate were collected using a heart rate monitor (Polar M200; Polar Electro Ltd) during all of the conditions.

#### Biomarkers

Cortisol level was measured immediately before and 4 hours after the interventions. Saliva samples were collected using cotton swabs (Salivette; Sarstedt Inc). Collected samples were stored in a freezer until the end of the study. The cortisol level in saliva will be measured by liquid chromatographic–tandem mass spectrometry. The analyses will be performed at the Hormone Laboratory at Oslo University Hospital.

Consistent with Salivette recommendations, participants were asked not to eat or drink, use tobacco, or brush their teeth 60 minutes before the saliva samples were taken.

### Exercise Interventions

The aerobic and anaerobic exercises included in the study were chosen on the basis of participant feedback given during the pilot study. All exercise sessions were supervised by a qualified staff member, a nurse with formal education in physical activity for people with mental health and addiction disorders.

#### Soccer

Soccer was played with 5 participants on each team, including the goalkeeper. It was arranged in gyms that the treatment centers normally use for physical activity sessions. The participants were randomly divided into 2 teams. If fewer than 10 patients participated in the study at one site, staff from that treatment center were asked to join. The field was 20 × 40 m and the goal was 3 × 2 m.

Participants were asked to play a friendly match where goals were counted. They were told their soccer competency was not important and that all participation in the match was valuable for the team, and they were asked to try to give it their best. The match lasted for 45 minutes, with a 2-minute break after 15 and 30 minutes.

#### Circuit Training

Circuit training was performed in the same gymnasium as the soccer intervention. Due to the low cost and limited need for equipment, we used body weight exercises only. Participants completed 4 circuits consisting of 8 individual exercise stations. They spent 40 seconds exercising at each station, then 20 seconds resting before transitioning to the next station. Between each circuit there was a 2-minute rest. As participants were new to the training environment, the first circuit took slightly longer to complete. Thus, the average duration of training for most participants was 45 minutes (42-48 minutes). The 8 exercises were air squats, inch worms, bench dips, frog jumps, sit-ups, push-ups, walking lunges, and back extensions.

During the first circuit, participants performed the exercises together to make sure they did them correctly. This circuit functioned as a warm-up and was undertaken at lower intensity. If a participant had a physical disability or was restricted from performing an exercise, an alternative or adjusted exercise was substituted. Participants were asked to give their best effort at each station and to perform as many repetitions as they could within 40 seconds. They were also told that if the exercises felt easy, they should try to increase the number of repetitions, whereas if they felt they were too strenuous, they could decrease the number.

### Control Condition

Participants attended a 45-minute lecture with a PowerPoint (Microsoft Corp) presentation on the benefits of physical activity for physical and mental health. The lecture format was similar to what the patients would normally receive during conventional treatment. The lecture was given by a member of the research group in a meeting room at the treatment centers.

### Adherence

A member of the research group was on-site during the interventions to remind participants about the assessment times and to motivate them to participate in the conditions. The same staff member was also available to answer any questions about the questionnaires.

### Statistical Analyses and Power Calculation

Statistical power was estimated using G*Power (version 3.1.9.7; Heinrich-Heine-Universität Düsseldorf). Parameters were based on results from our feasibility study [36] and also took into account previous studies demonstrating medium effect sizes for acute exercise on mood states [38,45]. Assuming an effect size of 0.3 and power (β) of .8, with 3 groups and 5 measurement points, we estimate needing at least 21 participants to test our hypotheses. We planned to include one group of 5 to 10 participants from each site, which would give us 20 to 40 participants. Due to the experimental study design and the inclusion of only inpatients, we anticipate minimal missing data. Descriptive data will be calculated for each measure. Changes over time will be assessed using mixed (group × time) repeated-measures analysis of variance with post hoc contrasts. If participants return questionnaires with missing data, the missing items will be imputed using the last observation carried forward method. If questionnaires are returned with more than 50% of data missing, the questionnaire will be excluded from the analysis.

### Safety, Data Management, and Confidentiality

During the trial, all patient data will be kept in a secure room accessible only by the research group. All data will be deidentified and treated according to the standards set by the Norwegian Data Inspectorate (Datatilsynet), in compliance with the Health Research Act and the Personal Data Act.

## Results

Data collection was completed in August 2019. In total, 39 patients admitted at 3 treatment centers participated in the study. Data will be analyzed during 2020.

## Discussion

Many SUD clinics have begun to implement physical activity as part of routine inpatient treatment due to its reported benefits. This study on acute exercise will add clinically relevant information about the short-term effects of soccer and circuit training on mood, craving, state anxiety, and self-esteem in this population. Most previous studies have performed pre-exercise and postexercise assessments only. The current study will add information on how the effects develop beyond the immediate cessation of exercise. The activities in the study are readily available, inexpensive, and popular in the general population. If the interventions are effective, they can easily be integrated into different clinical settings, for both inpatients and outpatients, and adapted to the participant’s fitness level.

The study is designed to reflect the clinical realities of inpatient treatment for SUDs. Typically, patients use multiple substances [3,35]. The inclusion of patients with comorbid SUD and mental disorders will give the study a high ecological validity but might also make the results difficult to interpret. Different drugs and various mental disorders might be affected in different ways by the interventions. Gathering information on the participants’ co-occurring disorders will potentially enable subgroup analyses to differentiate exercise effects in those with different combinations of substance and mental health problems.

Craving is highly relevant in clinical settings, even though the nature of it and the items included in instruments that assess craving have been debated [15,46,47]. By using a single-item scale, it will not be possible to capture changes in different aspects of craving. The participants might have different perceptions of craving, varying from a subjective desire to consume a substance to various bodily sensations. This is a potential limitation. However, previous studies assessing acute craving have used single-item VAS scales [48,49]. As the aim of the present study is to assess changes in participants’ overall subjective experience of craving and whether exercise can help attenuate it, we believe that a single-item scale can adequately capture this.

Using saliva samples is a noninvasive method for assessing cortisol levels. There is no need for medical staff, and saliva can be stored at room temperature for several hours before freezing. The drawback of saliva cortisol (versus serum cortisol) is that the use of tobacco and the presence of blood in the samples may lead to falsely elevated cortisol levels [50]. To control for this, the laboratory will not analyze samples with traces of blood. Samples with cortisol levels above reference levels will be excluded from analysis.

Dropout rates from treatment studies of SUDs are often high [6,28]. To minimize the risk of dropout, a member of the research group visited the treatment center 1 week before the study to answer questions. The recruitment took place a maximum of two weeks ahead of the trial. We tried to minimize dropout by keeping the overall intervention period short and integrating the interventions into the usual treatment routines at the treatment centers. This also minimized interference in the patients’ usual treatment.

## Abbreviations

CONSORT: Consolidated Standards of Reporting Trials
ICD-10: International Classification of Disease, Tenth Revision
PANAS: Positive and Negative Affect Schedule
RPE: Rating of Perceived Exertion
SPIRIT: Standard Protocol Items: Recommendations for Interventional Trials
SUD: substance use disorder
VAS: visual analog scale
